# Gene Expression Meta-Analysis of Seven Candidate Gene Sets for Diabetes Traits Following a GWAS Pathway Study

**DOI:** 10.3389/fgene.2018.00052

**Published:** 2018-02-16

**Authors:** Hao Mei, Lianna Li, Michael Griswold, Thomas Mosley

**Affiliations:** ^1^Department of Data Science, School of Population Health, University of Mississippi Medical Center, Jackson, MS, United States; ^2^Shanghai Children’s Medical Center, Shanghai Jiao Tong University School of Medicine, Shanghai, China; ^3^Department of Biology, Tougaloo College, Jackson, MS, United States; ^4^Department of Neurology, University of Mississippi Medical Center, Jackson, MS, United States

**Keywords:** pathway, gene expression, diabetes, meta-analysis, GWAS, enrichment analysis, association study

## Abstract

Seven gene sets were significantly enriched for SNP associations with diabetes, and considered as potential diabetes pathways in a previous meta-analysis of diabetes GWAS. This study aims to examine if these gene sets also have expression associations with diabetes. The analysis was conducted using pooled data from 23 diabetes gene expression studies. Gene associations were examined using linear modeling with an empirical Bayes approach, and pathway associations were investigated by testing enrichment for significant genes. Meta-analyses were performed to investigate gene and pathway associations in all studies and tissue types. The analysis showed that six gene sets and three member genes of ACADSB, RASSF2, and KLF12 had significant associations with diabetes traits. The findings suggest that these gene sets are related to diabetes regulation, and their functions tend to be tissue non-specific.

## Introduction

Diabetes mellitus is a chronic metabolic disease of hyperglycemia resulting from defects in insulin secretion, action, or both. The disease has two common etiologies: Type I diabetes (T1D) is mainly caused by beta-cell destruction, and Type II diabetes (T2D) is characterized by defects in insulin action and/or secretion. Development of diabetes is usually accompanied by varied symptoms and complications, and long-term diabetes may cause diabetic nephropathy, retinopathy, peripheral neuropathy and a range of cardiovascular symptoms (American Diabetes Association, 2014). Insulin regulation has been implicated in the pathogenesis of many of these diabetes traits.

Development of diabetes is affected by genetic predispositions within familial aggregations. The heritability of type I diabetes (T1D) is as high as 88% (Hyttinen et al., 2003). The concordance rate of type 2 diabetes (T2D) is 50–92% for monozygotic (MZ) twins, consistently greater than the rate for dizygotic (DZ) twins (Permutt et al., 2005). The complication of diabetic nephropathy has been observed in familial clustering (Seaquist et al., 1989). A twin bivariate genetic study of the Atherosclerosis Risk in Communities (ARIC) population showed that genetic heritability is 30% for fasting glucose and 39% for fasting insulin, and genetic correlation between them is 22 ∼ 39% (Vattikuti et al., 2012). Although T1D and T2D have different etiologies with pathologic changes observable in multiple tissues, they present some common clinical manifestations. A gene expression study has shown that both types of diabetes share pathogenic mechanisms and common pathways (Kaizer et al., 2007). Findings from these studies suggest that these diabetes traits have strong genetic susceptibility, and there are shared genetic components among them. Further genetic studies of diabetes traits and complications can help improve our understanding of the etiology and pathogenesis of diabetes.

Genome-wide association studies (GWAS) have identified hundreds of genetic risk variants associated with diabetes (Welter et al., 2014), typically single nucleotide polymorphisms (SNPs). In the post-GWAS era, ascertaining the biological roles of these variants underlying diabetes pathogenesis is essential. Investigations of expression quantitative trait loci (eQTL) have demonstrated that diabetes risk variants are linked to gene expression (Kang et al., 2012; van de Bunt et al., 2015), providing biological insight regarding variant associations and diabetes development. These linkages may be present in different tissues, showing non-specific characteristics. A genetic pathway, defined as a gene set, consists of genes related to specific biological functions. A diabetes pathway study of GWAS has suggested that SNP associations with diabetes traits are enriched in particular genetic pathways (Mei et al., 2015). Pathway investigations of gene expression have supplemented diabetes GWAS and eQTL studies that have been based on individual SNPs; this approach enhances our comprehension of how systemic genetic regulation may affect the etiology of diabetes. The broad application of advanced high-throughput technology in the past decade has led to the generation of abundant gene expression data sets collected from different tissue types, and gene expression studies can provide insight into genetic functions and biological mechanisms of diseases (Barrett et al., 2011). An investigation of pooled gene expressions from different studies and tissue types can help improve the power to identify gene and pathway signatures associated with a disease.

In our previous study, we proposed a novel method of uniform-score gene-set analysis (USGSA) that uses hypergeometric exact test statistic for GWAS-based pathway identification, and analysis of diabetes GWAS showed that seven gene sets are associated with diabetes traits and pathway member genes share common binding motifs for transcription factors (TFs) of FOXO4, NFAT, TCF3, VSX1 and POU2F1 and microRNA of MIR-218 (Mei et al., 2015). We extended the exact test of USGSA method with developed software package of *snpGeneSets* to generalize pathway analysis for both GWAS and gene expression study (Mei et al., 2016). Our analysis of NHGRI GWAS catalog and gene expression data using *snpGeneSets* evidenced that significant SNP associations and differential gene expressions of diabetes are enriched in particular pathways. We further performed pathway analysis of genome-wide differential gene expression and the results suggested that diabetes pathogenesis was involved with both tissue-specific and non-specific genetic pathways (Mei et al., 2017). Based on our studies and published findings, we hypothesize that diabetes pathways enriched for GWAS SNP associations are also enriched for differential gene expressions. We therefore conducted the present follow-up study to investigate our previously identified seven gene sets for their expression associations with diabetes traits in different tissues.

## Materials and Methods

### Gene Expressions of Candidate Gene Sets

The candidate pathways are seven gene sets enriched for GWAS SNP associations with diabetes traits identified our previous study (Mei et al., 2015) and annotated in the MSigDB database (Liberzon, 2014). The candidate pathways are summarized in **Table 1**, and each gene set was specified by its pathway ID (PID) (Mei et al., 2016). Member genes of every candidate gene set share common promoter binding motifs for particular transcription factors, including FOXO4, TCF3, NFAT, VSX1, and POU2F1 and microRNA of mir-218.

**Table 1:** Description of candidate gene sets.

PID	Gene set	Size	Motif	Binding
1461	AACTTT	1890	AACTTT	Unknown^1^
2247	FOXO4	2061	TTGTTT	FOXO4^2^
2268	NFAT	1896	TGGAAA	NFAT^3^
2240	TCF3	2485	CAGGTG	TCF3^4^
2076	MIR-218	398	AAGCACA	MIR-218^5^
2239	VSX1	810	TAATTA	VSX1^6^
1551	POU2F1	214	NNGAATATKCANNNN	POU2F1^7^

*PID, the pathway ID of gene set; Size, the number of member genes for candidate gene set; Motif, common promoter binding motif of member genes*.

*^*1*^http://www.broadinstitute.org/gsea/msigdb/cards/AACTTT_UNKNOWN*

*^*2*^http://www.broadinstitute.org/gsea/msigdb/cards/TTGTTT_FOXO4_01*

*^*3*^http://www.broadinstitute.org/gsea/msigdb/cards/TGGAAA_NFAT_Q4_01*

*^*4*^http://www.broadinstitute.org/gsea/msigdb/cards/CAGGTG_E12_Q6*

*^*5*^http://www.broadinstitute.org/gsea/msigdb/cards/AAGCACA_MIR218*

*^*6*^http://www.broadinstitute.org/gsea/msigdb/cards/TAATTA_CHX10_01*

*^*7*^http://www.broadinstitute.org/gsea/msigdb/cards/OCT1_02*

Gene expressions were obtained from the GEO data sets (GDS) (Barrett et al., 2011) for diabetes traits. All expression GDS were preprocessed and parsed through the R package of GEOquery (Davis and Meltzer, 2007). The *M*-value of each gene expression (i.e., log 2 -expression level) was extracted and normalized by quantile normalization using the *R* package, preprocessCore (Bolstad et al., 2003). Multiple GDS of the same samples were merged into a single data set, and the largest *M*-value was used as the study-specific gene expression (Dozmorov and Wren, 2011).

### Gene Expression Association and Meta-Analysis

Differential gene expression (DGE) was investigated by testing the null hypothesis that the expression *M*-value is equal across different diabetes trait statuses. The hypothesis was tested by fitting a linear model with the *R* package, limma (Smyth, 2004), which adjusts the standard errors by empirical Bayes approach to obtain an accurate *p*-value. The *snpGeneSets* package (Mei et al., 2016) was applied to calculate the association *U*-score and define significant genes. The association *U*-score for the *i*-th gene (*U*_i_) is calculated as U_i_ =(∑ _j_I (p_j_ < p_i_) + 0.5 ⋅∑ _j_I (p_j_ =p_i_))/N, where *p*_i_ is the *p*-value of the *i*-th gene and *N* is the total number of genes. The *U*-score approximately follows a uniform distribution, and genes with a *U*-score ≤ 0.05 were defined to have significant DGE.

Meta-analyses of DGE were conducted using both the binomial test and Fisher’s method over multiple expression studies. For a particular gene, the binomial test counted the number of studies with a *U*-score ≤ 0.05, and the meta-analysis *p*-value was gene_binp = pr (X ≥ 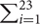
I (U_i_ < 0.05)), where *X* is a random variable following a binomial distribution. The Fisher’s method measured the gene’s combined *U*-scores over GDS, and the *p*-value was gene_fishp=Pr (X ≥-2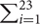 log (U_i_)), where *X* follows a chi-square distribution with *df* = 46. Study-specific DGE analysis and meta-analysis across studies were summarized in the Supplementary Figure 1.

### Pathway Expression Association and Meta-Analysis

The pathway expression association with diabetes traits was examined using the *snpGeneSets* package (Mei et al., 2016), which measures pathway enrichment of differential gene expressions by hypergeometric exact test. For a candidate gene set Ω and total *L* genes (*G*_i_, *i = 1,…,L*), the enrichment effect was measured as π_d_ =k/S - l/L, where K=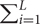
I (G_i_ ∈ Ω) I (U_i_ ≤ α), S=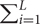
I (G_i_ ∈ Ω) and l=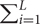
I (U_i_ ≤ α). The α equaled 0.05, defined as the significance level, and the estimate of π_d_ had a variation of _π_0_ (1-π_0_)/S_, approximately following a normal distribution. The pathway exact *p*-value was calculated as

$$path\_p=1-\sum_{i=0}^{K}\frac{\left(\begin{array}{c} S\\ i \end{array}\right)\left(\begin{array}{c} L-S\\ l-i \end{array}\right)}{\left(\begin{array}{c} L\\ l \end{array}\right)}$$

Besides the exact test of *snpGeneSets*, we also conducted test of pathway expression association with diabetes traits using different methods of gene set enrichment (Hung et al., 2012; Varemo et al., 2013), including Wilcoxon rank-sum and Fisher’s test from *piano* package (Varemo et al., 2013) and GSEA test (Subramanian et al., 2005) implemented in *fgsea* package (Sergushichev, unpublished), and obtained *p*-value based on 10,000 permutations.

Meta-analysis was conducted using binomial test to investigate pathway expression association over studies for all methods (Supplementary Figure 1), and binomial *p*-value was calculated as above. For exact test of *snpGeneSets*, the fixed-effect model was also applied to estimate pathway enrichment effect across studies, using the inverse of variance as a study-specific weight. The analysis was performed using the R package of *metaphor* (Viechtbauer, 2010).

### Pathway Expression Heterogeneity and KEGG Pathway Mapping

Pathway expression heterogeneity was tested using the McNemar’s chi-squared method, to compare significant expression patterns of a gene set between two studies (Supplementary Figure 1). For every pair of GDS studies, the number of inconsistent genes was counted, i.e., genes for which we found a significant DGE in one study but that were found to be insignificant in a second study. The *p*-value was adjusted by the Bonferroni method, and a finding of significant heterogeneity indicates that the gene set had a different expression pattern between studies.

The Kyoto Encyclopedia of Genes and Genomes (KEGG) (Kanehisa et al., 2010) pathway database is a compendium of known biological pathways related to human diseases. Our mapping analysis identified KEGG pathways statistically related to the candidate gene sets based on hypergeometric tests (Supplementary Figure 1). For a total of *L* genes (*G*_i_, *i = 1,…,L*), a candidate gene set Φ and a KEGG pathway Ω of *S* genes, the enrichment effect was measured as π_d_=k/S - l/L with a variation of _(l/L) (1-l/L)/S_, and the *p*-value was

$$p=1-\sum_{i=0}^{K}\frac{\left(\begin{array}{c} S\\ i \end{array}\right)\left(\begin{array}{c} L-S\\ l-i \end{array}\right)}{\left(\begin{array}{c} L\\ l \end{array}\right)},$$

where K=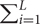
I (G_i_ ∈ Ω) I(G_i_ ∈ Φ) and l=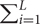
I(G_i_ ∈ Φ). An adjusted *p*-value was obtained using 10,000 permutation tests. The KEGG mapping analysis was conducted using the *snpGeneSets* package (Mei et al., 2016).

## Results

The procedures for gene and pathway expression analyses of the seven candidate gene sets, which are significantly enriched for GWAS SNP associations with diabetes traits (Mei et al., 2015), are summarized in the Supplementary Figure 1.

### Gene Expression Studies

Twenty-three expression studies, consisting of 30 GEO data sets (GDS) (Barrett et al., 2011), are summarized in **Table 2**. Diabetes traits explored in expression studies included insulin resistance, insulin sensitivity, T1D, T2D, diabetic nephropathy, diabetic neuropathy and diabetic heart failure (HF). Tissues used in expression assays included skeletal muscle, glomeruli, endothelial progenitor cells (EPCs), blood, adipocytes, and liver, heart, artery, nerve, myotube and pancreatic cells.

**Table 2:** Expression studies and GEO datasets.

Study	GDS_ID	Pub_ID	*N*_genes	Sample	Status	Tissue
1	**GDS157**	12436343	7129	10	Insulin resistant, insulin sensitive	Skeletal muscle
	**GDS158**		8934	10		
	**GDS160**		8924	10		
	**GDS161**		8928	10		
	**GDS162**		8928	10		
2	GDS961	15042541	12625	6	Diabetic nephropathy, normal	Glomeruli
3	GDS3656	19706161	24354	32	T1D, Healthy	EPC
4	GDS3665		32878	10	T2D, control	Adipocytes
5	GDS3681	18719883	12625	20	T2D, control	Myotube
6	GDS3782	20644627	61359	20	T2D, control	Pancreas
7	**GDS3874**	17595242	22283	117	Healthy, T1D, T2D	Blood
	**GDS3875**		22645			
8	GDS3876	19549744	22283	18	T2D, control	Liver
9	GDS3880	22802091	54675	42	T2D, pre-diabetes, normoglycemic control	Skeletal muscle
10	GDS3881	21423737	48702	22	Pre-surgery for T2D Obese, post-surgery for T2D Obese	Blood
11	GDS3882	21127054	22283	13	Non-diabetes, T2D	Pancreas
12	GDS3883	21035759	54675	17	T2D, normal glucose tolerance	Liver
13	GDS3884	21393865	54675	50	T2D, insulin resistant (FH-), insulin resistant (FH+)	Skeletal muscle
14	GDS3963	21829658	24526	24	Control, impaired fasting glucose, T2D	Blood
15	GDS3980	21926180; 22340758	22277	21	T2D, control	Artery
16	GDS4012	21926103	17788	35	Progressive, non-progressive diabetic neuropathy	Nerve
17	GDS4314	22427379	33297	24	Diabetic HF, non-diabetic HF	Heart
18	GDS4337	22768844	33297	63	T2D, non-diabetes	pancreas
19	**GDS2790**	17472435	7129	12	Baseline, insulin	Skeletal muscle
	**GDS2791**		22283			
20	GDS3104	17563058	54675	29	Healthy control, insulin resistance	Skeletal muscle
21	GDS3181	18334611	22283	36	-60, 30, and 240 min of hyperinsulinemia	Skeletal muscle
22	GDS3715	17709892; 21109598	12626	110	Insulin sensitive, insulin resistant, diabetes	Skeletal muscle
23	**GDS3781**	20678967	54675	39	Insulin sensitive, insulin resistant, diabetes	Adipocytes
	**GDS3962**			19		

*Study, the ID of expression study; GDS_ID, the ID of GEO data set; Pub_ID, PubMed ID of the study; N_genes, the number of genes assayed for expression. Bolded terms are the multiple datasets of a study.*

### Analysis of Gene Expression Association

**FIGURE 1:**
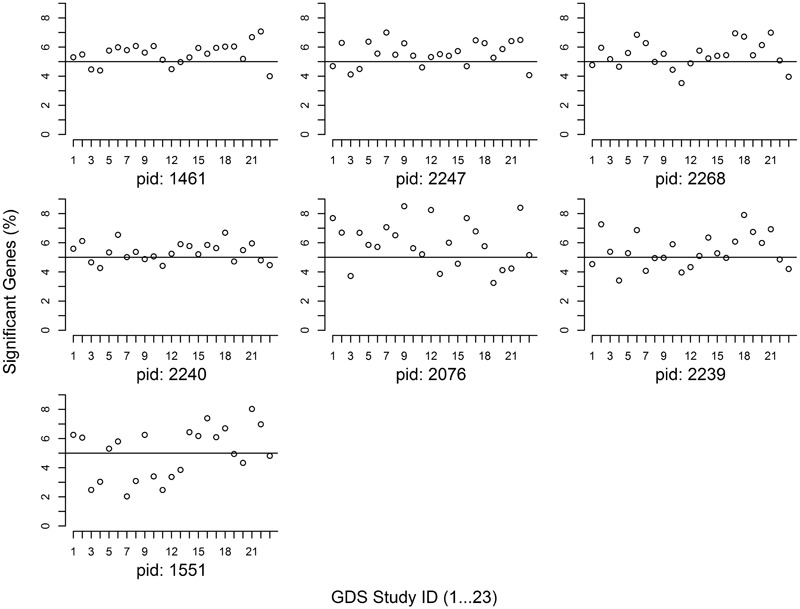
Proportion of significant genes at candidate gene sets. The study IDs in the *X-*axis are arrayed from 1 to 23. The *Y*-axis indicates the proportion of member genes with a *U*-score < 0.05. Pathway ID (PID): 1461 (AACTTT-motif), 2247 (FOXO4), 2268 (NFAT), 2240 (TCF3), 2076 (MIR-218), 2239 (VSX1), and 1551 (POU2F1).

The proportion of member genes with significant differential expression (i.e., an association *U*-score ≤ 0.05) is noted in **Figure 1** for every gene set. Given the null hypothesis that a pathway is not associated with a diabetes trait, it was expected that 5% of member genes would have significant expression associations. Our analysis showed that with the exception of the POU2F1 gene set (*PID: 1551*), more than 5% of member genes from all of the other 6 gene sets had significant expression associations.

Gene expression associations across studies were examined by meta-analysis using Fisher and binomial tests, and the *p*-values were calculated as *gene_fishp* and *gene_binp*, respectively. Three genes were found to be significant: (1) ACADSB (GID:36) belongs to the gene sets of VSX1 (PID:2239) and TCF3 (PID:2240) with *gene_fishp = 9.27^∗^10^-5^* and *gene_binp = 9.71^∗^10^-6^*; (2) RASSF2 (GID:9770) belongs to the gene sets TCF3 (PID:2240), AACTTT–motif (PID:1461), FOXO4 (PID:2247) and NFAT (PID:2268) with *gene_fishp = 1.32^∗^10^-4^* and *gene_binp = 9.71^∗^10^-6^*; and 3) KLF12 (GID: 11278) belongs to the gene set of MIR-218 (PID:2076) with *gene_fishp = 5.65^∗^10^-4^* and *gene_binp = 9.4^∗^10^-6^* (see **Table 3**). The negative log 10 of each *U*-score and the corresponding Bayes *p*-value are shown in Supplementary Figure 2.

**Table 3:** Meta-analysis of gene expression associations with diabetes traits.

GID	Gene	Chr	Start (BP)	End (BP)	Strand	PID	gene_fishp	gene_binp
36	ACADSB	10	124768429	124817806	+	2240;2239	9.27E-05	9.71E-06
9770	RASSF2	20	4760669	4804291	-	1461; 2247; 2268; 2240	1.32E-04	9.71E-06
11278	KLF12	13	74260149	74708400	-	2076	5.65E-04	9.40E-05

*GID: the NCBI gene ID. PID (pathway ID): 1461 (AACTTT-motif), 2247 (FOXO4), 2268 (NFAT), 2240 (TCF3), 2076 (MIR-218), and 2239 (VSX1). gene_fishp: meta-analysis p-value by Fisher’s method; gene_binp: meta-analysis p-value by binomial test.*

### Analysis of Pathway Expression Association

**FIGURE 2:**
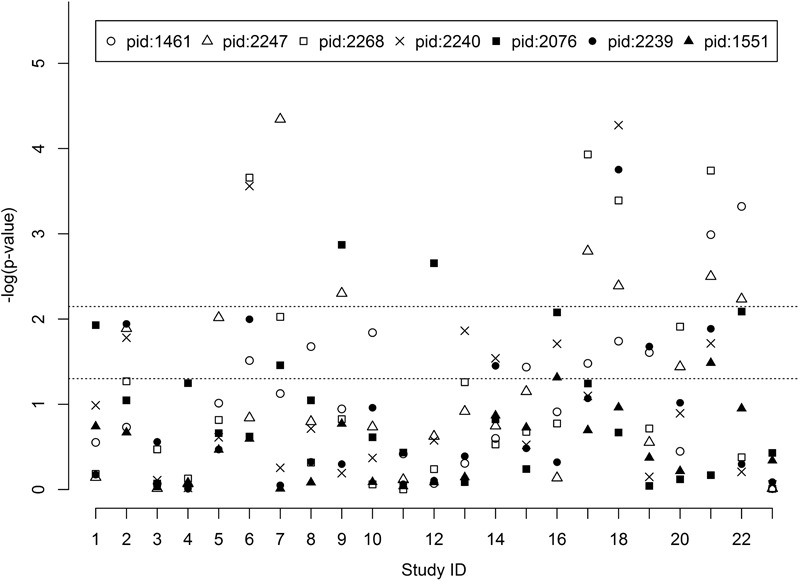
Pathway expression association of candidate gene sets. The study IDs in the *X*-axis are arrayed from 1 to 23. The *Y*-axis indicates the -log_10_ (*p*-value) of every candidate gene set. Pathway ID (PID): 1461 (AACTTT-motif), 2247 (FOXO4), 2268 (NFAT), 2240 (TCF3), 2076 (MIR-218), 2239 (VSX1) and 1551 (POU2F1).

Study-specific pathway *p*-values with different methods are shown in Supplementary Figures 3a–g. The hypergeometric exact test of *snpGeneSets* (Exact), Wilcoxon, Fisher and GSEA methods, respectively, had 28.0, 37.9, 37.9, and 28.6% of tests with *p*-value≤0.05. The results suggest that different methods have consistent pathway association pattern, and the Exact had over 75% of concordant tests with the other three methods. Study-specific pathway *p*-values of hypergeometric exact test by *snpGeneSets* for each gene set is shown in **Figure 2**. The significance level, based on Bonferroni adjustment, was *p* = 0.007. The gene sets of FOXO4 (PID: 2247), NFAT (PID: 2268) and VSX1 (PID: 2239) were found to have significant expression associations in *6, 4*, and *1* studies, respectively, and the gene sets of AACTTT-motif (PID: 1461), TCF3 (PID: 2240) and MIR-218 (PID: 2076) each were found to have significant expression associations in two studies. There was no significant expression association for POU2F1 (PID: 1551). The FOXO4 and TCF3 gene sets both had the minimum *p*-values of *4.5^∗^10^-5^* and *5.3^∗^10^-5^* in Study 7, which measured gene expressions in blood samples from healthy, T1D and T2D individuals, and in Study 18, which measured expressions in pancreatic tissue samples from T2D and non-diabetic individuals, respectively.

Meta-analysis *p*-values of seven candidate gene sets were calculated by binomial test to measure their associations with diabetes traits across studies for all four methods (Supplementary Table 1). The meta-analysis suggested that these methods had consistent test results: all gene sets, except the POU2F1 (PID: 1551), presented significant meta-analysis *p*-values of pathway expression associations. The results also showed that the exact test of *snpGenesets* had conservative meta-analysis *p*-value compared to the other three methods.

The exact test of *snpGeneSets* provides estimate of pathway enrichment effect and calculation of association *p*-value. Meta-analysis of pathway expression associations with diabetes traits was conducted using binomial and fixed-effect tests to measure effects over studies, which yielded *p*-values of *path_binp* and *path_fixp*, respectively. Results are summarized in **Table 4** and plotted in Supplementary Figures 4a–g. Except for POU2F1 (PID:1551), all other six gene sets had strong significant associations with diabetes traits after Bonferroni correction (*p*-value < 0.007). MIR-218 (PID:2076) had the largest effect of π_d_ = 0.94% with a *95%* confidence interval of [0.46, 1.42], and *p*-values of *path_fixp = 1.22^∗^10^-4^* and *path_binp = 9.4^∗^10^-5^*. FOXO4 (PID:2247) had the smallest *p*-values of *path_fixp = 4.94^∗^10^-6^* and *path_binp = 6.11^∗^10^-8^*. AACTTT-motif (PID:1461), NFAT (PID:2268), TCF3 (PID:2240) and VSX1 (PID:2239) had *path_fixp* (*path_binp*) of *4.08^∗^10^-5^* (*6.11^∗^10^-8^*), *6.79^∗^10^-5^* (*9.40^∗^10^-5^*), *7.14^∗^10^-4^* (*9.71^∗^10^-6^*), and *0.02* (*9.40^∗^10^-5^*), respectively.

**Table 4:** Meta-analysis of pathway expression associations with diabetes traits.

PID	Effect (%)	*SE*	*Z*	CI_LB	CI_UB	path_fixp	path_binp
1461	0.46	0.11	4.10	0.24	0.68	4.08E-05	6.11E-08
2247	0.49	0.11	4.57	0.28	0.70	4.94E-06	6.11E-08
2268	0.45	0.11	3.98	0.23	0.67	6.79E-05	9.40E-05
2240	0.33	0.10	3.38	0.14	0.53	7.14E-04	9.71E-06
2076	0.94	0.25	3.84	0.46	1.42	1.22E-04	9.40E-05
2239	0.41	0.17	2.38	0.07	0.75	0.02	9.40E-05
1551	-0.08	0.34	-0.22	-0.73	0.58	0.82	0.11

*PID (pathway ID): 1461 (AACTTT-motif), 2247 (FOXO4), 2268 (NFAT), 2240 (TCF3), 2076 (MIR-218), 2239 (VSX1), and 1551 (POU2F1). CI_LB and CI_UB: lower and upper bound of 95% confidence interval; path_fixp: meta-analysis p-value by the fixed-effect model; path_binp: meta-analysis p-value by binomial test.*

Stratified meta-analysis of pathway expression association with T2D based on exact test of *snpGeneSets* was performed over 11 studies and 7 tissues with results summarized in the Supplementary Table 2. Consistent with diabetes traits, the POU2F1 gene set (PID:1551) is insignificant (*p*-value > 0.05), while all other six gene sets have *p*-value < 0.05 in meta-analysis of binomial test. Besides, gene sets of FOXO4 (PID:2247) and TCF3 (PID:2240) have *p*-values < 0.007 in both binomial and fixed-effect tests; AACTTT-motif (PID:1461) and VSX1 (PID:2239) gene sets have *p*-values of *path_binp = 1.12^∗^10^-4^* and *0.002*, respectively; and the MIR-218 gene set has *p*-value of *path_fixp = 0.002*. Pathway analysis of expression association with T1D in the EPCs (Study 3) did not find any significant candidate gene set (*p*-value > 0.05). Pathway analysis of T1D association in the blood mononuclear cells (Study 7) showed that gene sets of AACTTT-motif (PID:1461), FOXO4 (PID:2247), NFAT (PID:2268), MIR-218 (PID:2076) and VSX1 (PID:2239) have *p*-values of *3.95^∗^10^-4^, 7.05^∗^10^-6^, 0.04, 0.04*, and *0.06*, respectively (Supplementary Table 3).

### Analysis of Expression Heterogeneity and Pathway Mapping

We performed *253* pairwise heterogeneity tests of pathway expression patterns for 23 expression studies, and the significant *p*-value based on Bonferroni adjustment was ≤*2E-4*. The negative log 10 of each *p*-value for the heterogeneity tests is plotted in Supplementary Figure 5. Ten significant tests were identified, accounting for 0.6% of all comparisons. The FOXO4 gene set (PID:2247) showed significantly different expression patterns for Study 7 (blood) vs. Study 3 (endothelial progenitor cells) and Study 23 (adipocytes). NFAT (PID:2268) had eight significant heterogeneity tests—for Study 23 (adipocytes) vs. Study 6 (pancreas), Study 17 (heart) and Study 21 (skeletal muscle); and for Study 11 (pancreas) vs. Study 6 (pancreas), Study 7 (blood), Study 17 (heart), Study 18 (pancreas), and Study 21 (skeletal muscle). These results suggest that the candidate gene sets have tissue-specific expression associations with diabetes traits. However, significant heterogeneity of pathway expressions was not found in the majority of studies and tissues (>99%), which indicates that the candidate gene sets tend to have consistent expression patterns for different diabetes traits and tissues.

KEGG pathway mapping of the candidate gene sets found effects and *p*-values ranging from 5.5% ∼ 23.4% and *5.7E-04 ∼ 3.63E-11*, respectively (see **Table 5**). The AACTTT-motif gene set (PID: 1461) was mapped to four signaling pathways of Wnt, MAPK, Insulin and TGF-beta with an adjusted *p*-value (*adj_p*) ≤ *3E-04*. The FOXO4 gene set (PID: 2247) was mapped to MAPK (*adj_p = 6.1E-03*) and TGF-beta (*adj_p < 1E-04*) signaling pathways. The NFAT gene set (PID: 2268) was mapped to Wnt (*adj_p = 0.01*), MAPK (*adj_p < 1E-04*) and TGF-beta (*adj_p = 5E-04*) signaling pathways. The TCF3 gene set (PID: 2240) was mapped to Wnt and MAPK signaling pathways with *adj_p < 1E-04*. The MIR-218 gene set (PID: 2076) was mapped to the Axon guidance pathway with *adj_p = 0.041*. Finally, the VSX1 gene set (PID: 2239) was mapped to the tight junction pathway with *adj_p = 0.016*. The POU2F1 gene set (PID: 1551) was not significantly mapped to any KEGG pathways.

**Table 5:** KEGG pathway mapping of candidate gene sets.

PID	KEGG	Effect (%)	*SE*	*p*	adj_p
1461	WNT^1^	19.4	2.5	3.63E-11	<1E-4
	MAPK^2^	11.8	1.9	1.01E-08	<1E-4
	INSULIN^3^	13.8	2.7	3.12E-06	3E-04
	TGF^4^	18.0	3.4	3.18E-06	3E-04
2247	TGF	23.4	3.4	7.60E-09	<1E-4
	MAPK	8.0	1.9	6.25E-05	6.1E-03
2268	MAPK	11.6	1.9	2.38E-08	<1E-4
	TGF	17.8	3.4	4.77E-06	5E-04
	WNT	10.6	2.6	1.13E-04	0.01
2240	MAPK	13.0	2.2	1.14E-08	<1E-4
	WNT	17.1	2.9	5.34E-08	<1E-4
2076	Axon guidance	5.5	1.3	5.7E-04	0.041
2239	Tight junction^5^	7.6	1.8	1.9E-4	0.016

*PID (pathway ID): 1461 (AACTTT-motif), 2247 (FOXO4), 2268 (NFAT), 2240 (TCF3), 2076 (MIR-218), and 2239 (VSX1); ^1^WNT, Wnt signaling pathway (http://software.broadinstitute.org/gsea/msigdb/cards/KEGG_WNT_SIGNALING_PATHWAY); ^2^MAPK, MAPK signaling pathway (http://software.broadinstitute.org/gsea/msigdb/cards/KEGG_MAPK_SIGNALING_PATHWAY); ^3^INSULIN, Insulin signaling pathway (http://software.broadinstitute.org/gsea/msigdb/cards/KEGG_INSULIN_SIGNALING_PATHWAY); ^4^TGF, TGF-beta signaling pathway (http://software.broadinstitute.org/gsea/msigdb/cards/KEGG_TGF_BETA_SIGNALING_PATHWAY); and ^5^Tight junction pathway (http://software.broadinstitute.org/gsea/msigdb/cards/KEGG_TIGHT_JUNCTION)*.

## Discussion

We investigated 7 GWAS-identified gene sets for their expression associations with diabetes traits in 23 independent studies. Our analysis found that 6 gene sets had a higher-than-expected proportion of significant member genes with differential expressions (**Figure 1**), suggesting potential correlations between gene expression regulation and diabetes risk DNA variants. In contrast to our previous hypothesis-free pathway expression study of diabetes traits (Mei et al., 2017), this meta-analysis of candidate gene sets following GWAS helps to expand our understanding of biology function of GWAS findings.

Member genes of the candidate gene sets share particular promoter motifs bound by transcription factors of FOXO4, NFAT, TCF3, VSX1, POU2F1, or microRNA of MIR-218. We applied two types of meta-analysis to test gene expression association with diabetes traits across studies (Supplementary Figure 1): the binomial method was based on the number of significant study-specific DGE tests, and the Fisher’s method was conducted using the combined *p*-values of individual studies. Both meta-analyses presented consistent tests of DGE and the results showed that genes of ACADSB, RASSF2, and KLF12 had significant gene expression associations with diabetes traits (**Table 3**). ACADSB is a member gene of the TCF3 and VSX1 gene sets, and it encodes the mitochondrial enzyme involved in metabolism of fatty acids (Rozen et al., 1994). The RASSF2 gene belongs to gene sets of AACTTT-motif, FOXO4, NFAT, and TCF3, and it encodes the proteins of the RAS family, which are important intracellular signal transducers regulating various biologic processes. KLF12 is a component gene of the MIR-218 gene set, and its protein represses expression of AP-2 alpha transcription factor (Roth et al., 2000), a developmentally regulated activator of transcription. These genes are all highly expressed in blood, and their significant expression associations in different tissues suggest that the encoded proteins may be potential biomarkers for measuring diabetes risk.

We applied four methods of pathway enrichment test to study expression association of candidate gene sets with diabetes traits. All methods showed consistent patterns of pathway association tests and the exact test of *snpGeneSets* presented more conservative *p*-values than the other three methods. In contrast to the methods of Fisher, Wilcoxon and GSEA, the hypergeometric exact test of *snpGeneSets* also provides estimate of pathway enrichment effect in addition to calculation of pathway association *p*-value. We used two types of meta-analysis to estimate pathway enrichment effect and association *p*-value for pathway expression association with diabetes traits over studies: the fixed-effect model test was dependent on the combined effects of all studies and the binomial method was based on the number of significant individual studies. Both types of meta-analysis showed consistent results for the seven candidate gene sets. Our analyses with different methods and meta-analyses provide a relatively comprehensive evaluation of pathway expression association with diabetes traits.

The pathophysiology of diabetes and its complications typically involve diverse tissues. A prior gene expression study has indicated that T1D and T2D share common pathways related to hyperglycemia and beta-cell dysfunctions (Kaizer et al., 2007). Our stratified meta-analysis of expression studies indicated that the pathophysiology of diabetes involves tissue non-specific pathways. For example, the FOXO4 gene set was found to be significant in blood, skeletal muscle, heart, myotube, glomeruli and pancreatic tissues (**Figure 2** and Supplementary Figure 4b), and more than 99% of our heterogeneity analyses found no significant difference in expression patterns across studies and tissues (Supplementary Figure 5). We also observed that 0.6% of tests had significantly different expression patterns across tissues, e.g., expressions of the FOXO4 gene set between blood (study 7) and adipocytes (study 23), indicating that the candidate gene sets also have tissue-specific roles in diabetes pathogenesis. Compared to meta-analysis of diabetes traits over all expression studies, the stratified meta-analysis of pathway expression association with T2D among 11 studies and 7 tissues presented almost consistent results for the 7 candidate gene sets (Supplementary Table 2). For pathway analysis in the two expression studies of T1D, we did not observe any significant association in the study 3 of endothelial progenitor cells, while analysis in the study 7 of blood mononuclear cells identified two gene sets with strongly significant association (*p*-value < 0.001), two gene sets with *p*-value < 0.05 and one with *p*-value = 0.06 (Supplementary Table 3). The results suggest that some candidate gene sets are not associated with T1D and some have pathway expression associations at particular tissues.

We also performed a mapping analysis to infer the functions of candidate gene sets. The mapped KEGG pathways suggest that the gene sets are involved in the pathogenesis of diabetes (**Table 5**). The Wnt (Jin, 2008), MAPK (Zhang et al., 2011), TGF-beta (Yadav et al., 2011) and insulin signaling pathways are known to regulate insulin sensitivity, lipid metabolism, glucose and energy homeostasis; the axon guidance pathway is associated with Wnt proteins (Zou, 2004); and the tight junction pathway is related to the pathophysiology of diabetes (Harhaj and Antonetti, 2004). The results from our mapping analyses are also consistent with previous findings: miR-218 has a regulation effect by stimulating the Wnt pathway (Hassan et al., 2012); activation of the NFAT factor is calcium-dependent, and hypocalcemia is associated with impaired insulin secretion (Pittas et al., 2007); the FOXO4 factor binds to insulin-response elements, and insulin can induce phosphorylation of FOXO4 and inhibit FOXO4-dependent gene transcription (Kops et al., 1999).

In recent years, GWAS has been widely applied to identify risk variants associated with diabetes, but it remains challenging to explain how these variants affect diabetes traits. We believe that this GWAS follow-up study will help to address the challenge and may play an important role in improving our understanding of the potential biological mechanisms of DNA variants that underlie diabetes pathogenesis. Our expression meta-analysis suggests that diabetes-associated variants are related to gene and pathway expression associations, which is consistent with previous investigations of expression quantitative trait loci (eQTLs) based on GWAS risk SNPs of diabetes, shown to influence gene expressions (Kang et al., 2012). A recent study that investigated enrichment of functional elements for GWAS-identified variants across tissues, including eQTL, protein binding sites, enhancers and promoters, observed that although a few comparisons were significantly tissue-specific for these functional elements, most tests tend to be tissue non-specific (Markunas et al., 2017). The findings are consistent with our pathway heterogeneity tests across tissues, in which only 0.6% of comparisons had statistically significant tissue-specific expression patterns. In contrast to these studies focused on functional roles of individual GWAS risk variants, our study was conducted to investigate expression associations between diabetes traits and pathways enriched for GWAS-identified SNP associations. We believe that our findings can be used to guide follow-up functional studies of GWAS SNPs and genetic pathways underlying diabetes pathogenesis.

Our study is not without limitations. All expression studies in the current meta-analysis were based on the microarray platform, which includes less transcriptome detail than is provided by next-generation sequencing. In addition, our meta-analysis was performed on curated GDS of gene expressions identified from the GEO that have a few limitations: (1) most diabetes studies are for T2D, with only two for T1D; (2) all expression datasets have a relatively small sample size (≤117); and (3) many tissue types were collected in only 1–3 studies. These limitations can affect the reliability of statistical tests and reduce the study power. It will be worthwhile to conduct replication studies on more expression studies with larger sample sizes and different tissue types in a future extension of the current meta-analysis. In addition, identification of tissue-non-specific gene and pathway expression associations were based on statistical tests which do not provide direct evidence for the roles of genes and pathways in diabetes pathogenesis. Therefore, future *in vivo* biological studies of these genes and pathways will be essential in improving our understanding of the genetic regulation mechanisms of diabetes.

In summary, we investigated expression associations of 7 candidate gene sets and their member genes with diabetes traits in 23 expression studies. Our analysis showed that these gene sets, enriched for SNP associations with diabetes traits, were also enriched for differential gene expressions, and the pathway expression pattern tended to be tissue-non-specific. The mapping analysis of KEGG pathways identified potential regulation mechanisms of these gene sets underlying the pathogenesis and development of diabetes. We believe that our findings will facilitate the discovery of novel regulation pathways for understanding diabetes genetics, and will advance the study of clinical biomarkers for early prevention and diagnosis of diabetes.

## Author Contributions

HM and LL designed the experiment, carried out the analyses and prepared the manuscript. MG and TM prepared and revised the manuscript.

## Conflict of Interest Statement

The authors declare that the research was conducted in the absence of any commercial or financial relationships that could be construed as a potential conflict of interest.
