# Supplementary material for: Gene Expression Meta-Analysis of Seven Candidate Gene Sets for Diabetes Traits Following a GWAS Pathway Study
Source: Front Genet. 2018 Feb 16;9:52. doi: 10.3389/fgene.2018.00052 (PMC5820295; doi:10.3389/fgene.2018.00052)
Supplement: Supplementary file 1 [file Presentation_1.PDF]

Supplementary Table 1: Meta-analysis p-values of pathway expression association with diabetes traits

PID	Exact	Wilcoxon	Fisher	GSEA
1461	6.11E-08	3.25E-13	3.25E-13	1.96E-10
2247	6.11E-08	1.96E-10	3.25E-13	6.11E-08
2268	9.40E-05	6.11E-08	3.76E-09	9.71E-06
2240	9.71E-06	3.76E-09	6.11E-08	9.71E-06
2076	9.40E-05	9.71E-06	9.40E-05	9.40E-05
2239	9.40E-05	9.71E-06	8.39E-07	7.54E-04
1551	0.11	4.93E-03	0.11	0.32

Meta-analysis p-values were calculated from binomial test of pathway expression association based on different methods. Exact: hypergeometric exact test of *snpGeneSets*; Wilcoxon: Wilcoxon rank-sum test of *piano*; Fisher: Fisher's combination test of *piano*; GSEA: the GSEA test of *fgsea*.

Supplementary Table 2. Stratified meta-analysis of pathway expression associations with T2D

PID	Effect (%)	S.E.	Z	CI_LB	CI_UB	path_fixp	path_binp
1461	0.38	0.16	2.34	0.06	0.69	0.02	1.12E-04
2247	0.51	0.15	3.28	0.20	0.81	0.001	0.002
2268	0.39	0.16	2.39	0.07	0.71	0.02	0.02
2240	0.44	0.14	3.08	0.16	0.71	0.002	1.12E-04
2076	1.12	0.35	3.17	0.43	1.81	0.002	0.02
2239	0.33	0.25	1.33	-0.002	0.82	0.18	0.002
1551	-0.22	0.48	-0.46	-1.17	0.72	0.65	0.43

Stratified meta-analysis of pathway expression association with T2D based on exact test of *snpGeneSets* was conducted in Study 4 (GDS3665), 5 (GDS3681), 6 (GDS3782), 8 (GDS3876), 9 (GDS3880), 11 (GDS3882), 12 (GDS3883), 13 (GDS3884), 14 (GDS3963), 15 (GDS3980) and 18 (GDS4337). PIDs (pathway IDs) of 7 candidate gene sets with are: 1461 (AACTTT-motif), 2247 (FOXO4), 2268 (NFAT), 2240 (TCF3), 2076 (MIR-218), 2239 (VSX1) and 1551 (POU2F1). CI_LB and CI_UB: lower and upper bound of 95% confidence interval; path_fixp: meta-analysis p-value by the fixed-effect model; path_binp: meta-analysis p-value by binomial test.

Supplementary Table 3. Pathway expression associations with T1D

PID	Effect (%)	S.E.	p-value
1461	1.72	0.51	3.95E-04
2247	2.15	0.49	7.05E-06
2268	0.93	0.52	0.04
2240	0.35	0.45	0.21
2076	2.07	1.12	0.04
2239	1.31	0.79	0.06
1551	1.60	1.55	0.19

Pathway expression association with T1D based on exact test of *snpGeneSets* was conducted in the Study 7. PID (pathway ID): 1461 (AACTTT-motif), 2247 (FOXO4), 2268 (NFAT), 2240 (TCF3), 2076 (MIR-218), 2239 (VSX1) and 1551 (POU2F1).

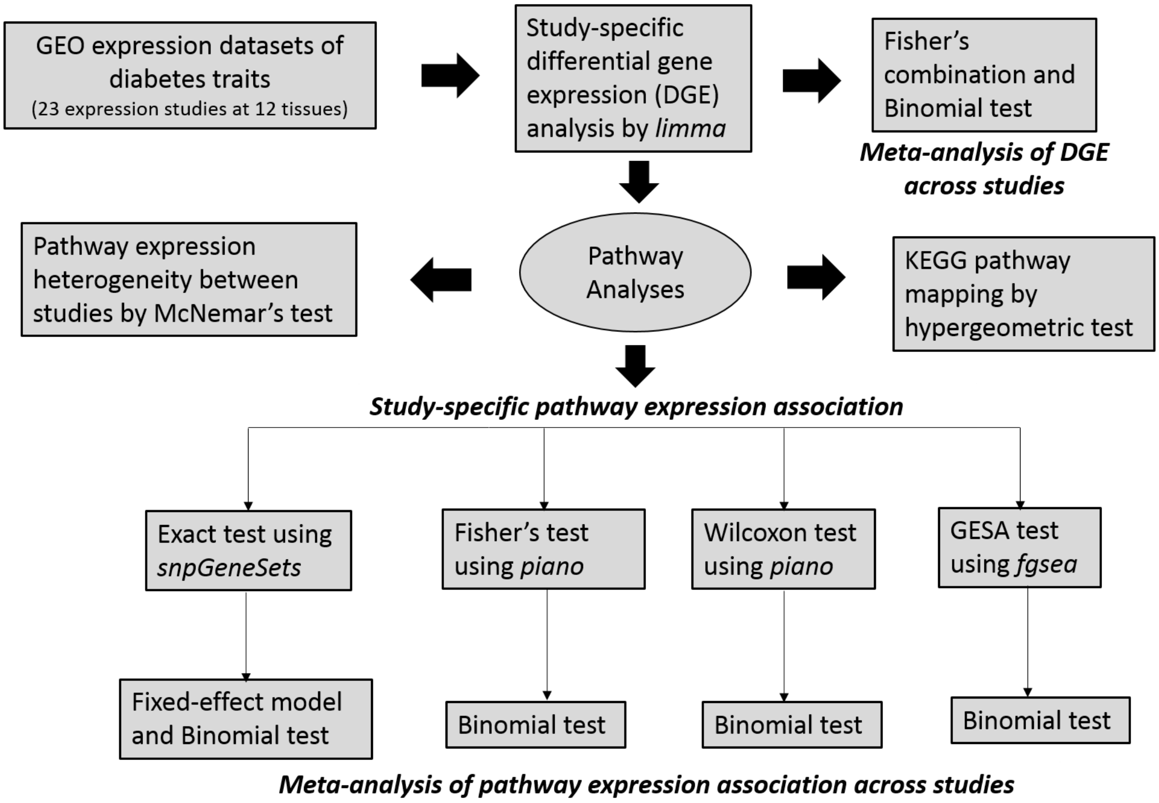

Supplementary Figure 1. Gene and pathway expression analyses of 7 candidate gene sets

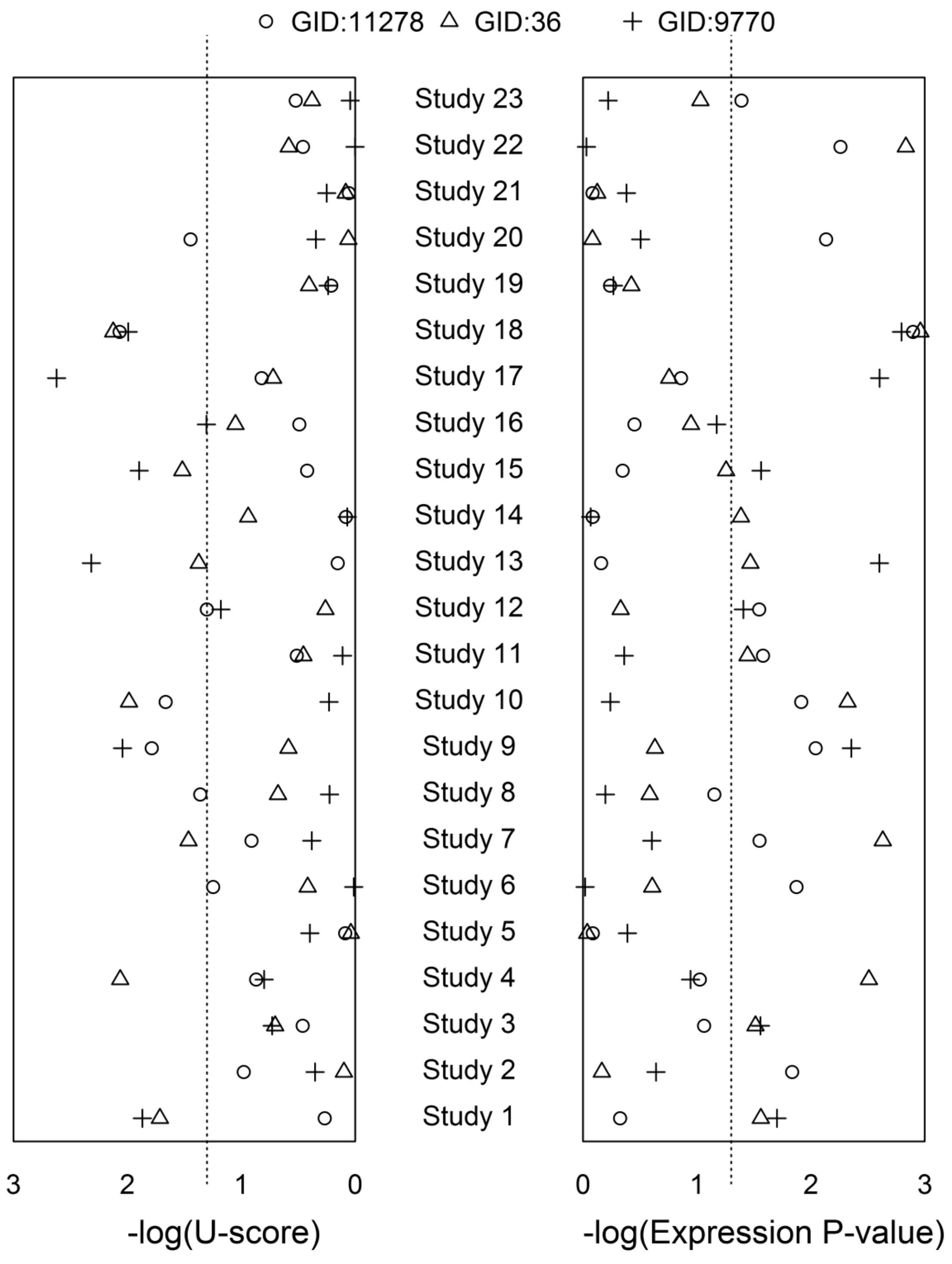

Supplementary Figure 2. Negative log 10 of U-score and Bayes p-value

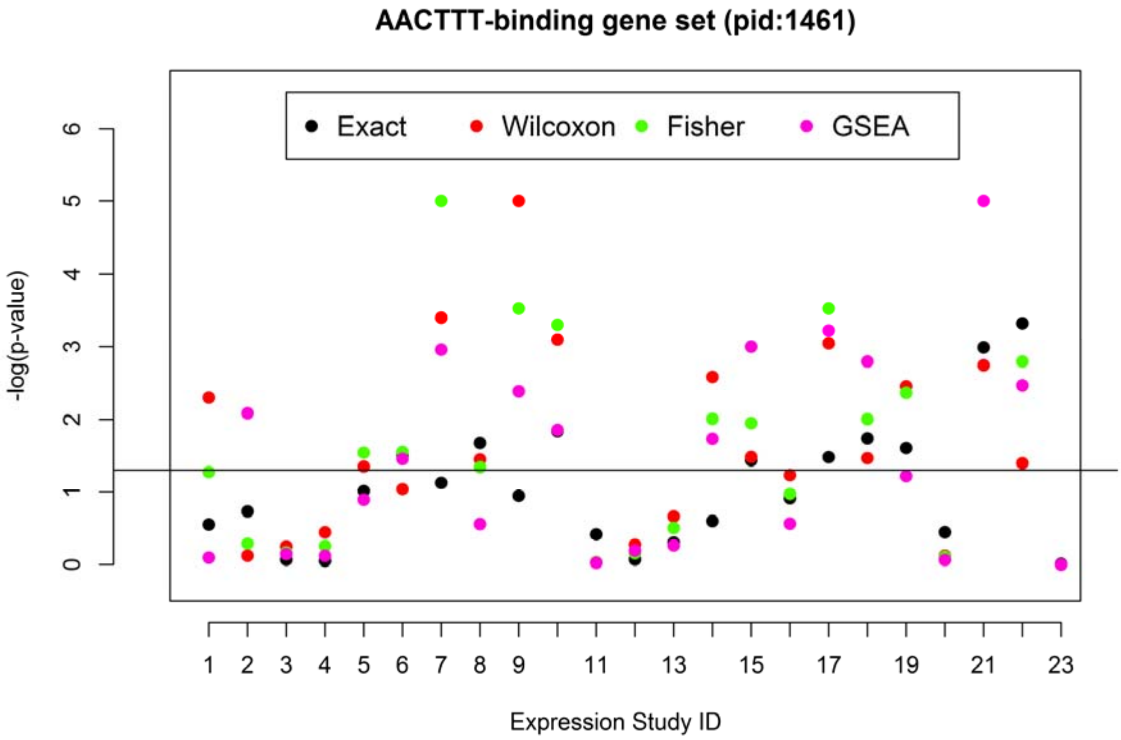

Supplementary Figure 3a. Study-specific pathway expression association of AACTTT-binding gene set (pid:1461) by 4 different methods of enrichment analysis. Exact: Hypergeometric exact test using *snpGeneSets*; Wilcoxon: Wilcoxon rank-sum test using *piano*; Fisher: Fisher's combination test using *piano*; and GSEA: GSEA test using *fgsea*.

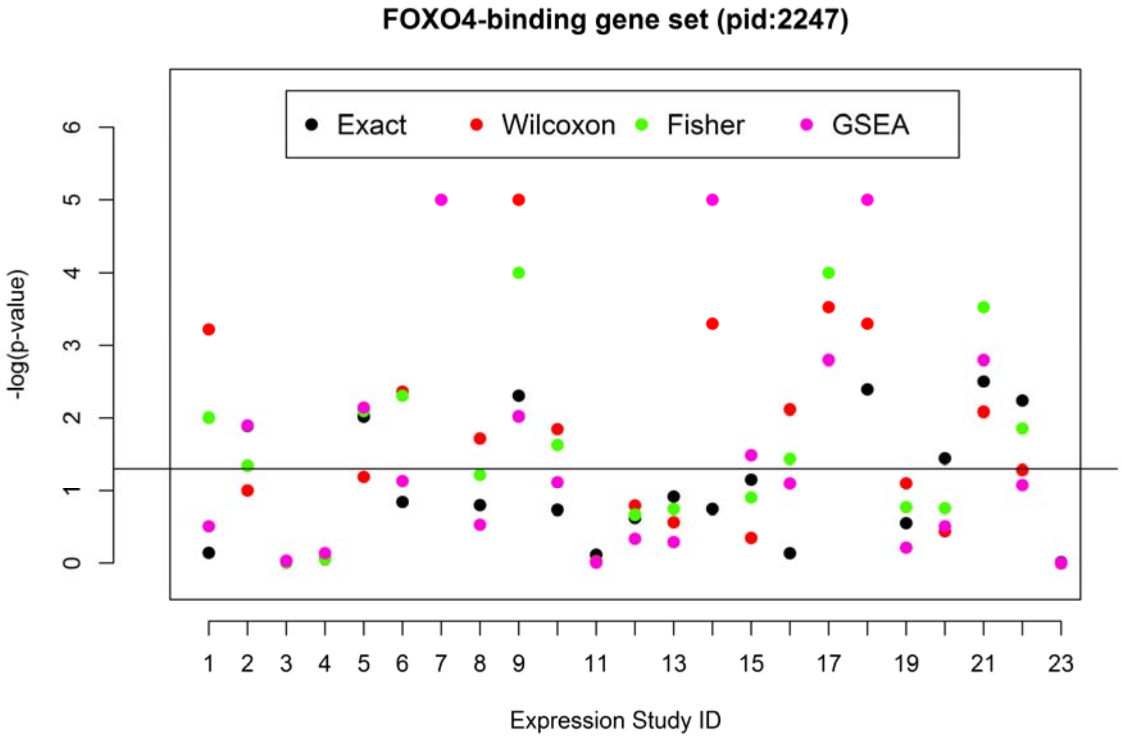

Supplementary Figure 3b. Study-specific pathway expression association of FOXO4 -binding gene set (pid:2247) by 4 different methods of enrichment analysis. Exact: Hypergeometric exact test using *snpGeneSets*; Wilcoxon: Wilcoxon rank-sum test using *piano*; Fisher: Fisher's combination test using *piano*; and GSEA: GSEA test using *fgsea*.

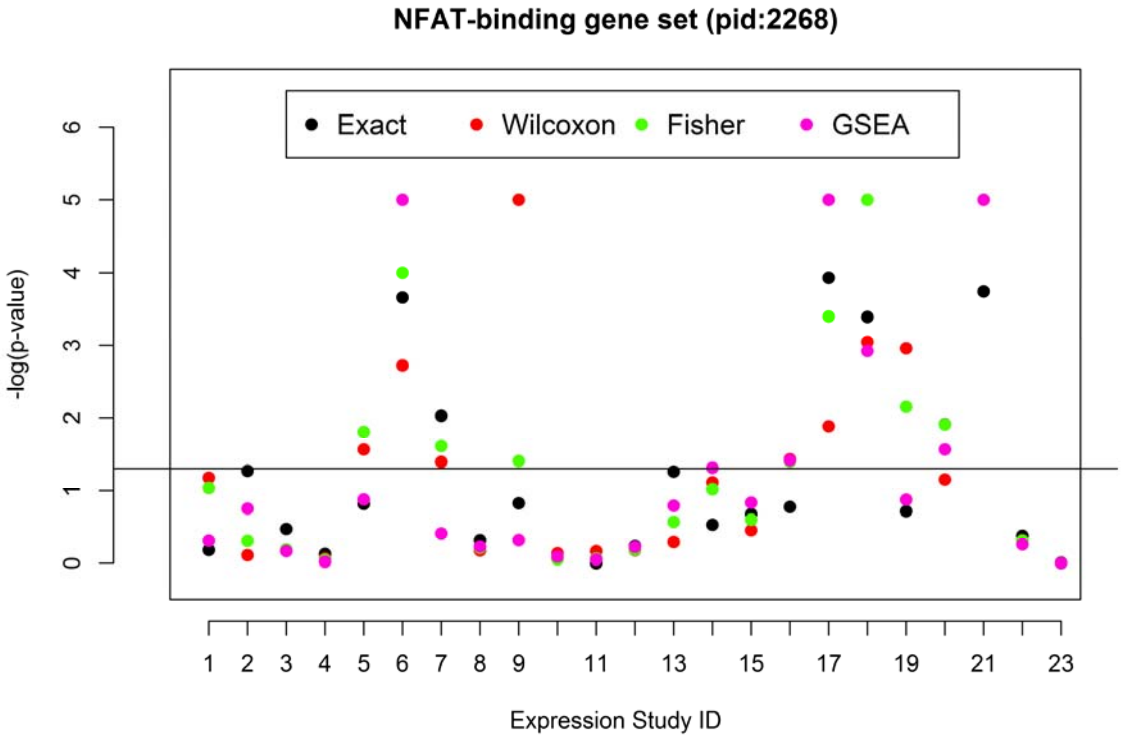

Supplementary Figure 3c. Study-specific pathway expression association of NFAT - binding gene set (pid:2268) by 4 different methods of enrichment analysis. Exact: Hypergeometric exact test using *snpGeneSets*; Wilcoxon: Wilcoxon rank-sum test using *piano*; Fisher: Fisher's combination test using *piano*; and GSEA: GSEA test using *fgsea*.

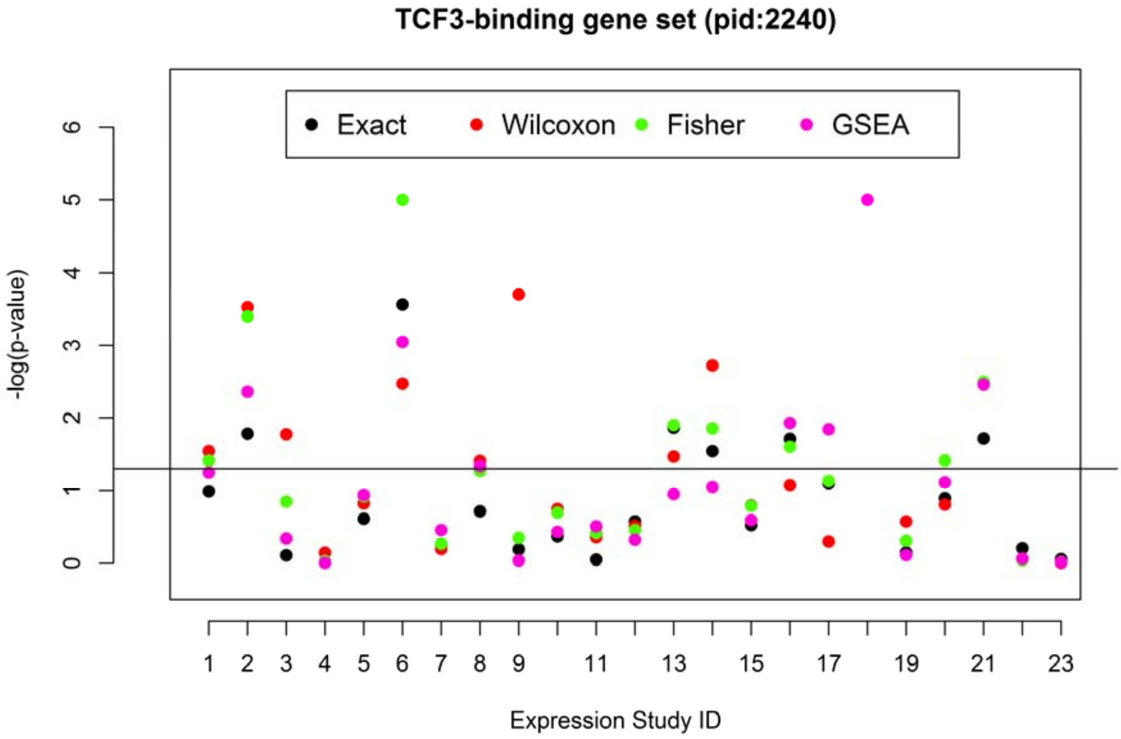

Supplementary Figure 3d. Study-specific pathway expression association of TCF3 - binding gene set (pid:2240) by 4 different methods of enrichment analysis. Exact: Hypergeometric exact test using *snpGeneSets*; Wilcoxon: Wilcoxon rank-sum test using *piano*; Fisher: Fisher's combination test using *piano*; and GSEA: GSEA test using *fgsea*.

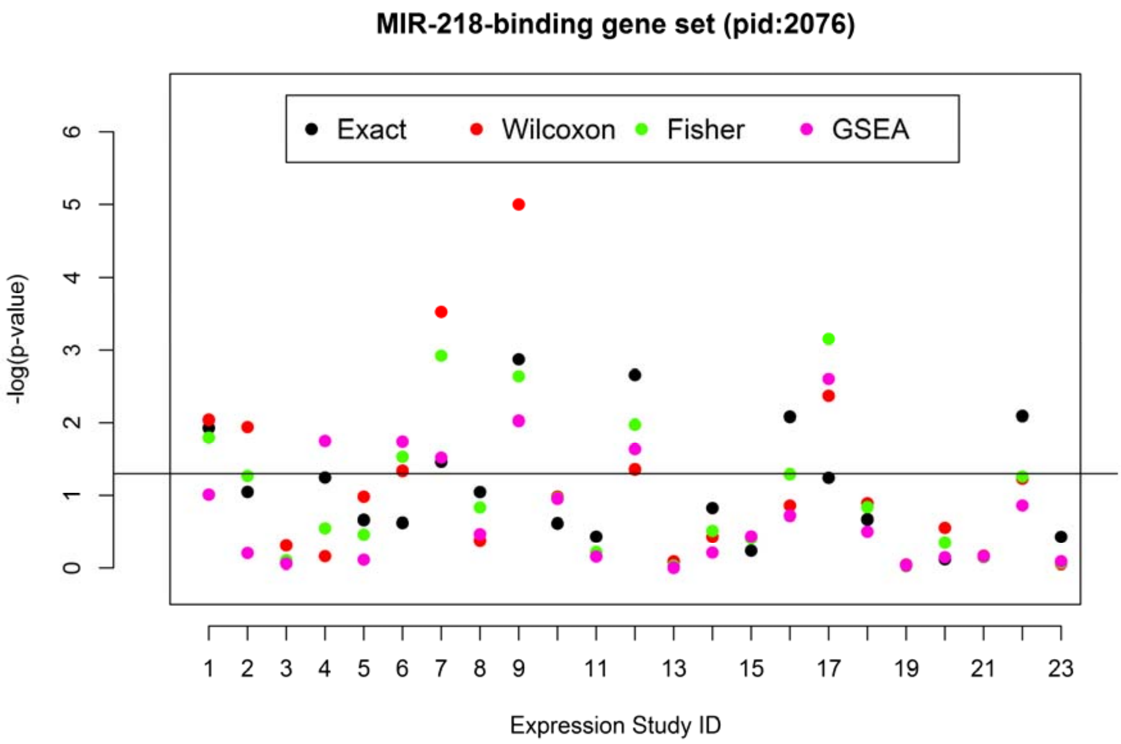

Supplementary Figure 3e. Study-specific pathway expression association of MIR - 218 - binding gene set (pid:2076) by 4 different methods of enrichment analysis. Exact: Hypergeometric exact test using *snpGeneSets*; Wilcoxon: Wilcoxon rank-sum test using *piano*; Fisher: Fisher's combination test using *piano*; and GSEA: GSEA test using *fgsea*.

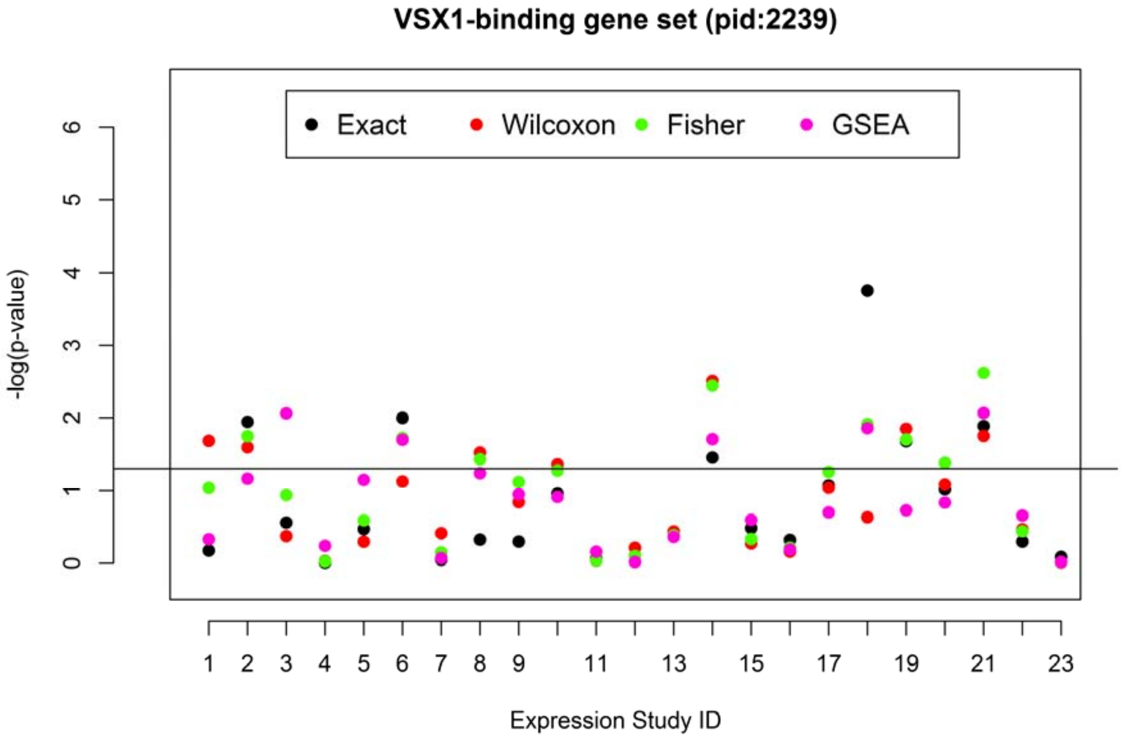

Supplementary Figure 3f. Study-specific pathway expression association of VSX1 - binding gene set (pid:2239) by 4 different methods of enrichment analysis. Exact: Hypergeometric exact test using *snpGeneSets*; Wilcoxon: Wilcoxon rank-sum test using *piano*; Fisher: Fisher's combination test using *piano*; and GSEA: GSEA test using *fgsea*.

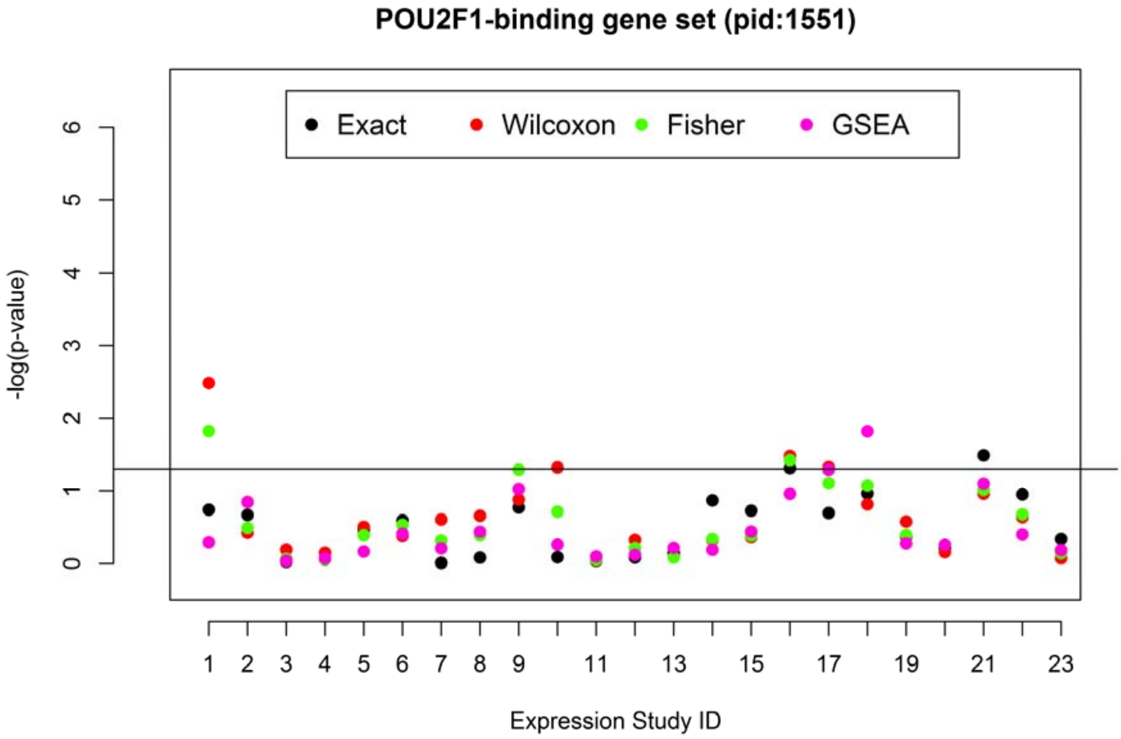

Supplementary Figure 3g. Study-specific pathway expression association of POU2F1 - binding gene set (pid:1551) by 4 different methods of enrichment analysis. Exact: Hypergeometric exact test using *snpGeneSets*; Wilcoxon: Wilcoxon rank-sum test using *piano*; Fisher: Fisher's combination test using *piano*; and GSEA: GSEA test using *fgsea*.

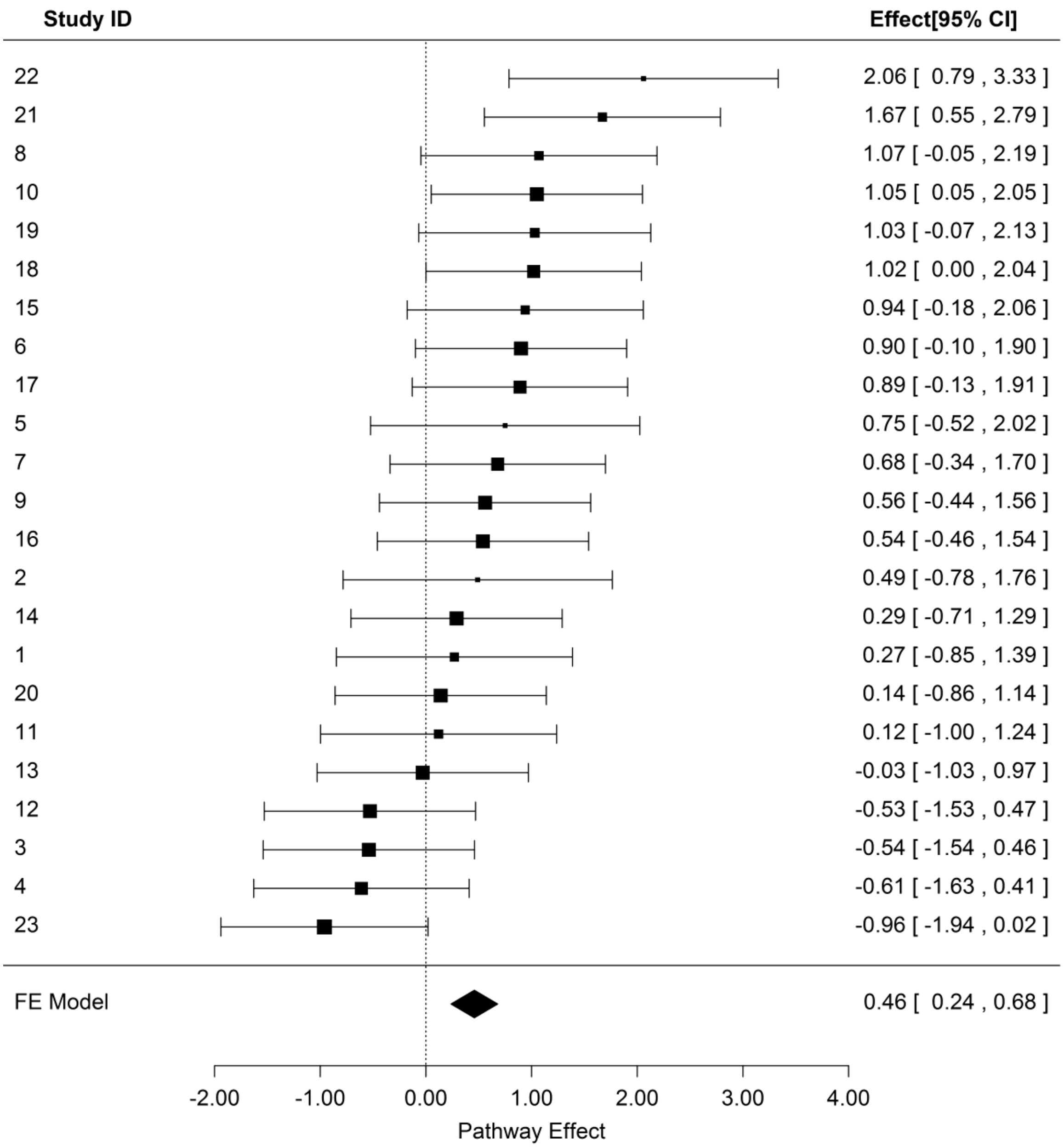

Supplementary Figure 4a. Meta-analysis of AACTTT-binding gene set (pid:1461)

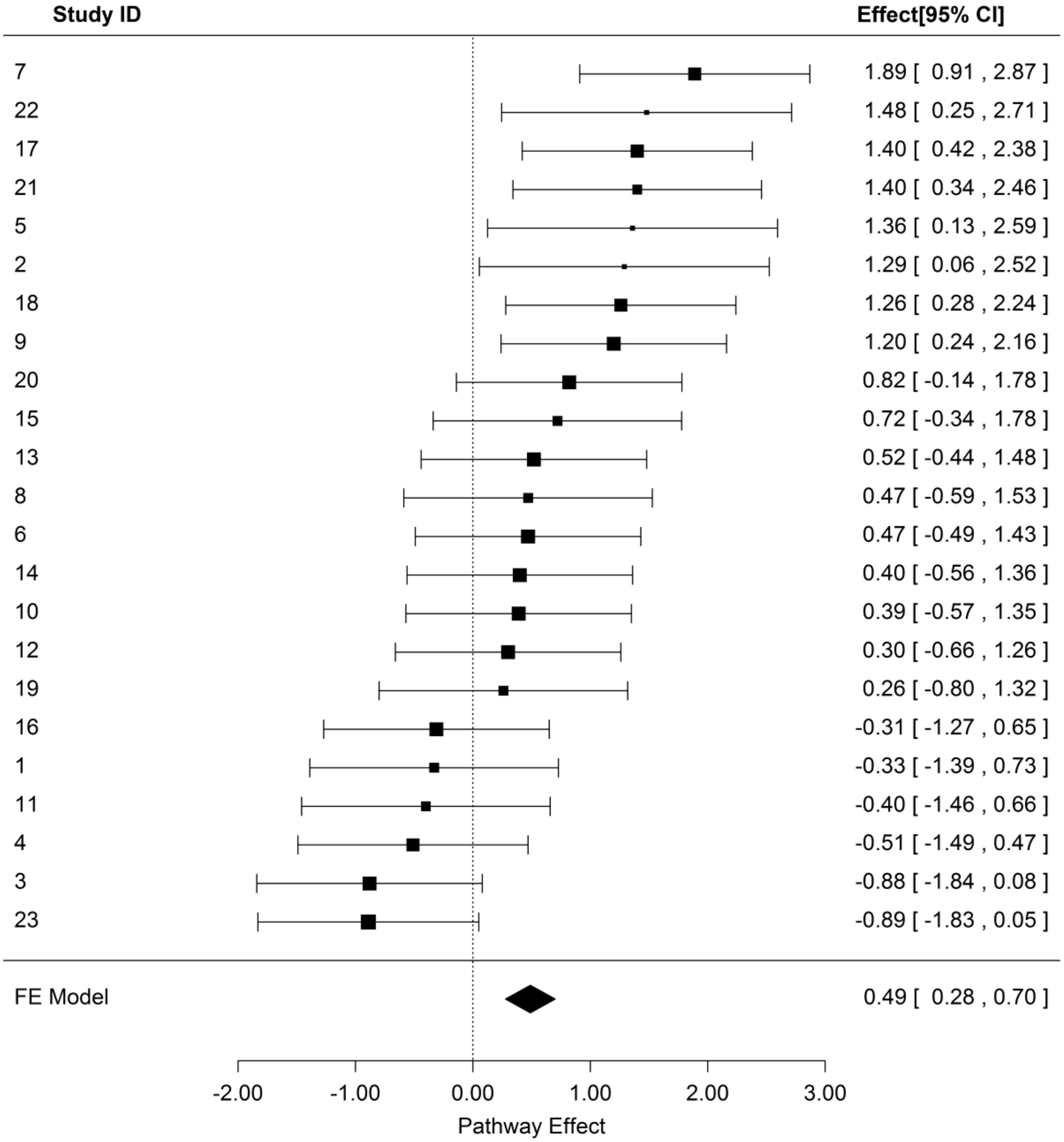

Supplementary Figure 4b. Meta-analysis of FOXO4-binding gene set (pid:2247)

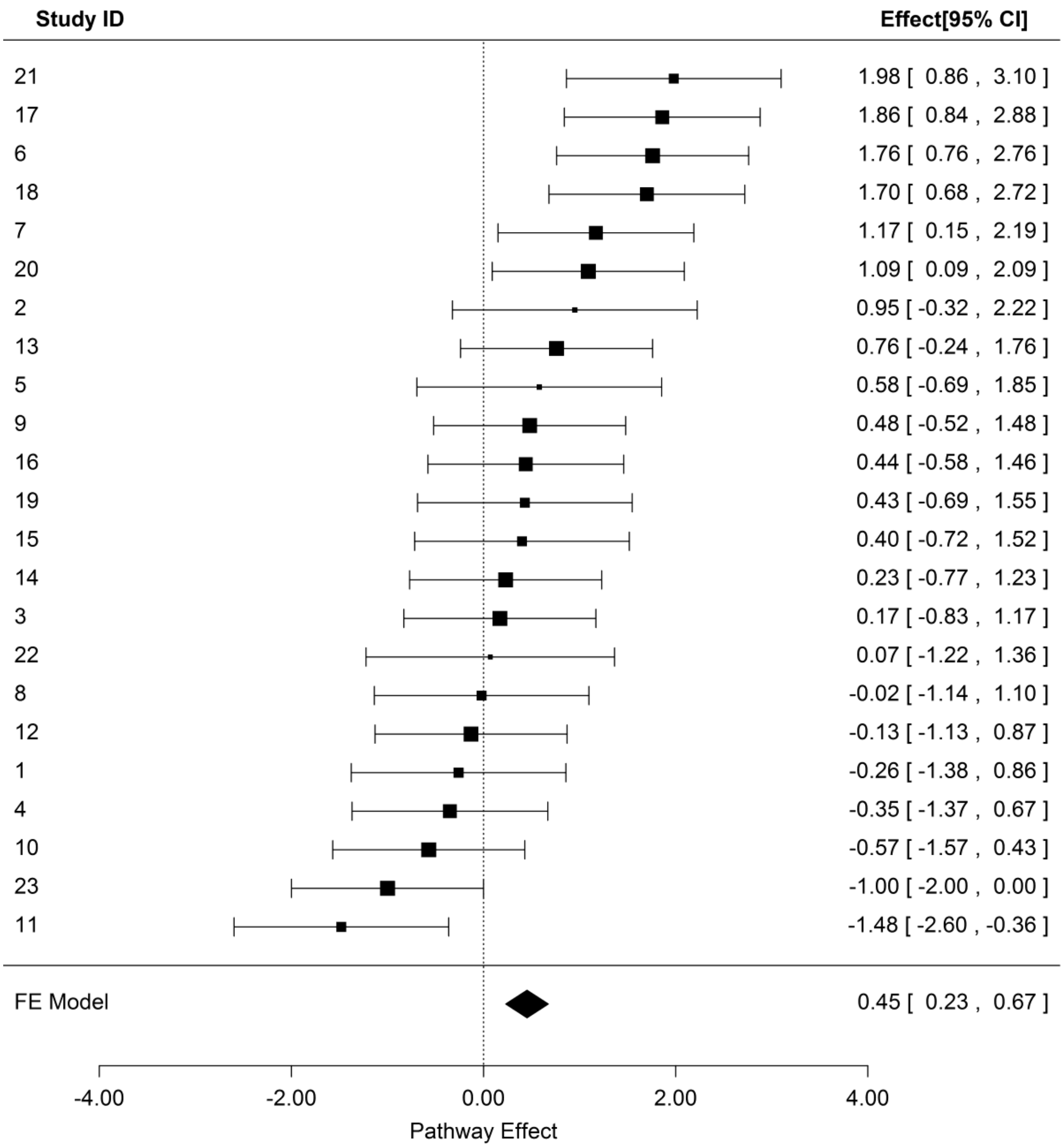

Supplementary Figure 4c. Meta-analysis of NFAT-binding gene set (pid:2268)

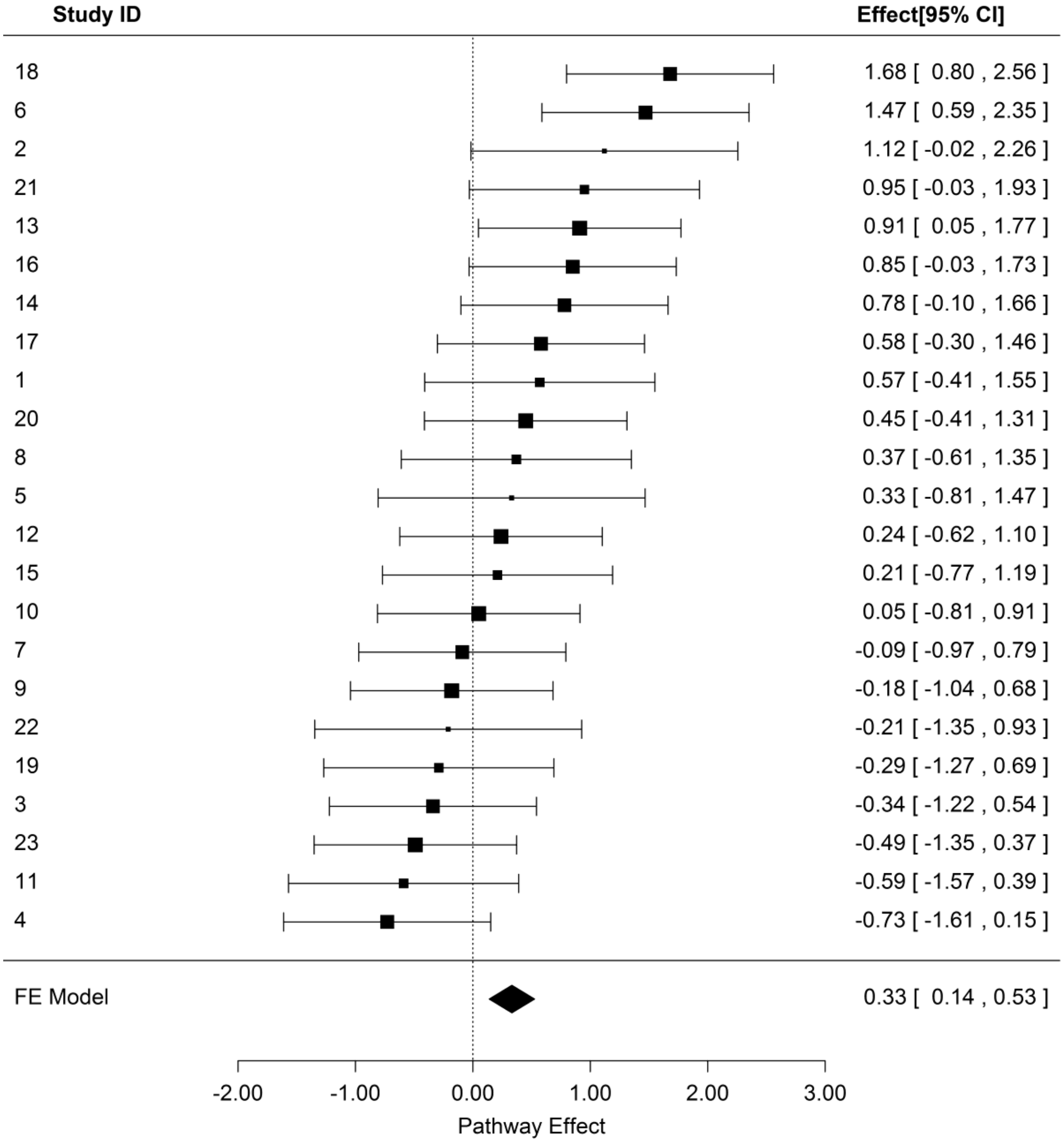

Supplementary Figure 4d. Meta-analysis of TCF3-binding gene set (pid:2240)

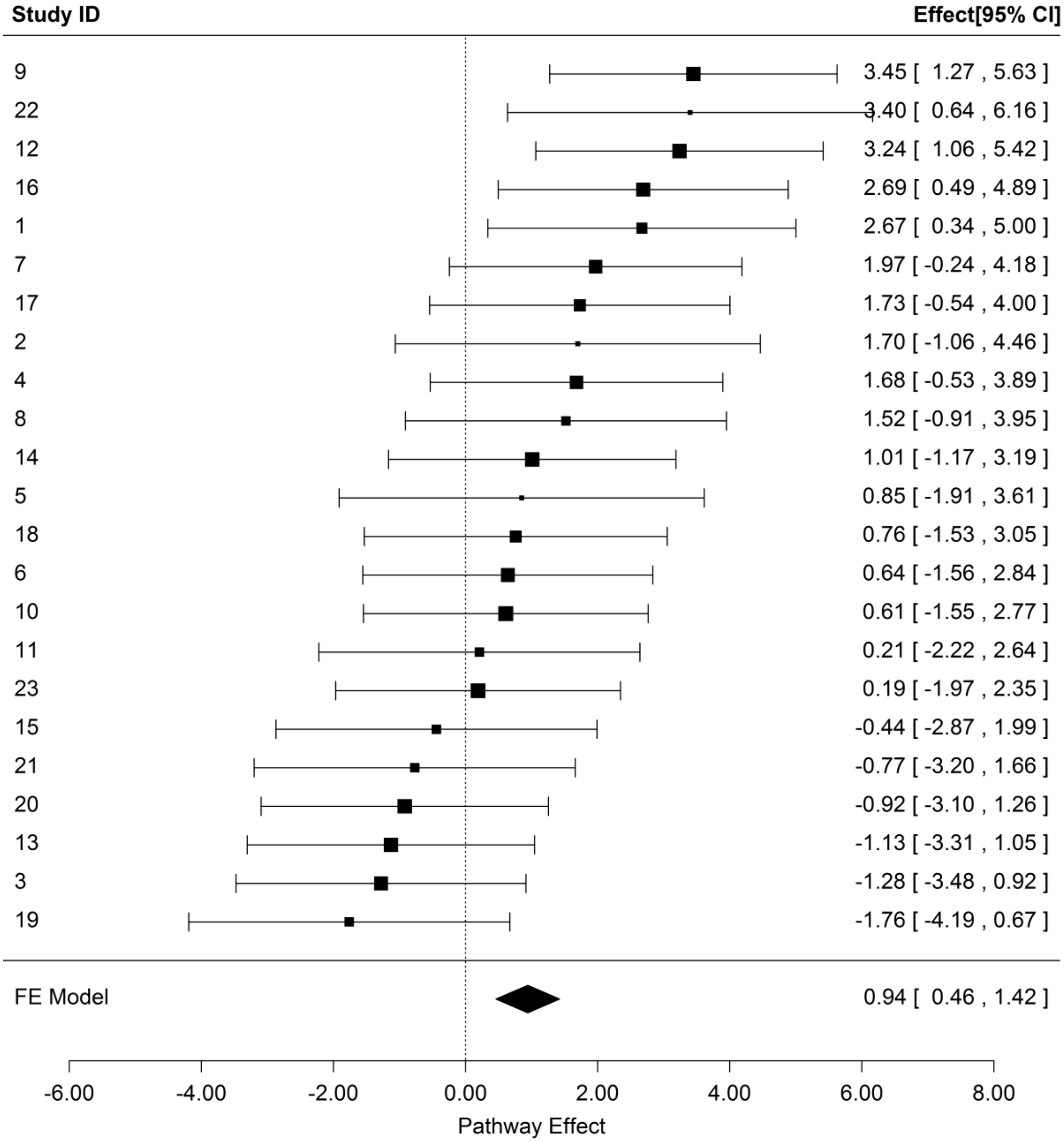

Supplementary Figure 4e. Meta-analysis of MIR-218-binding gene set (pid:2076)

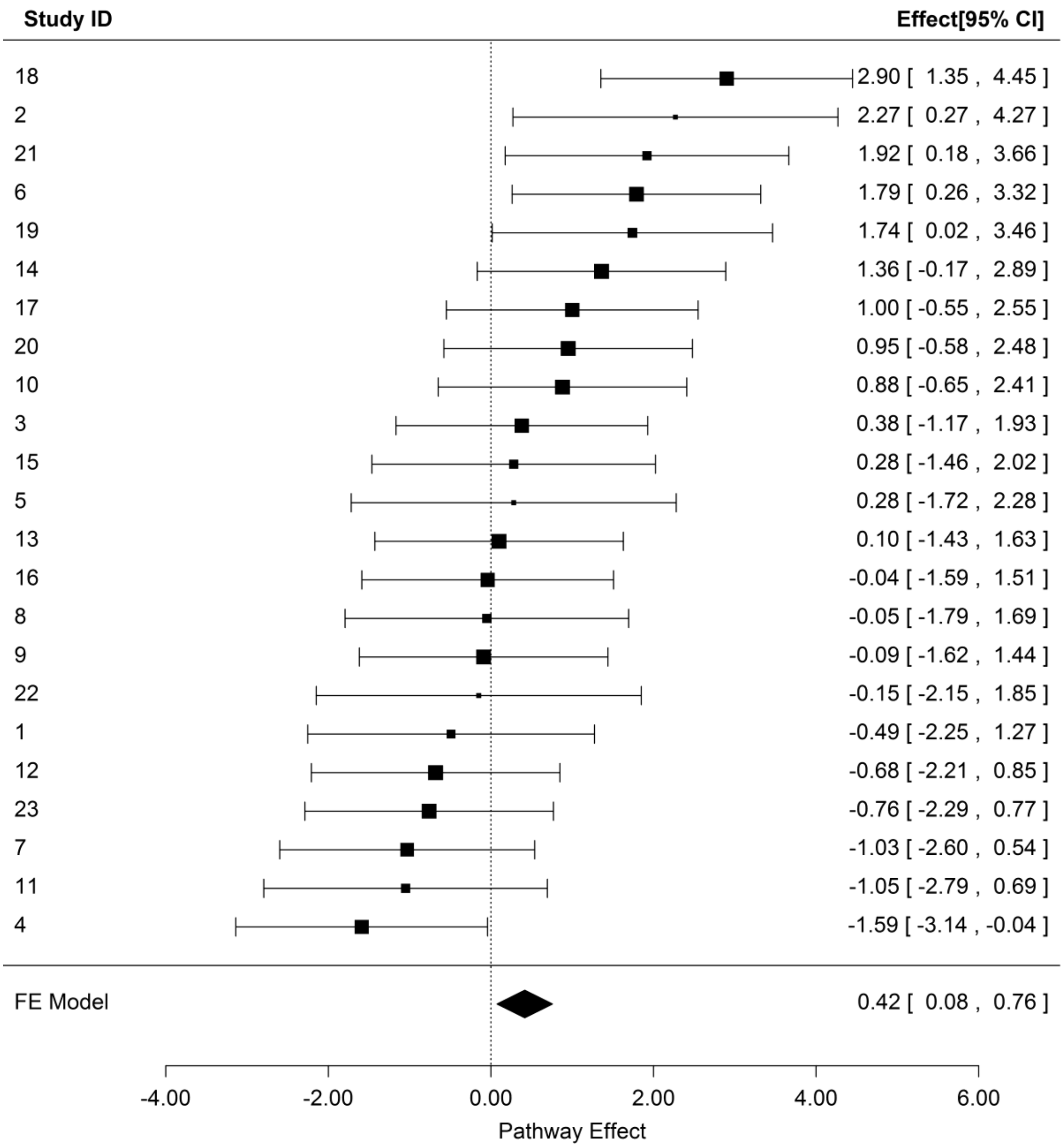

Supplementary Figure 4f. Meta-analysis of VSX1-binding gene set (pid:2239)

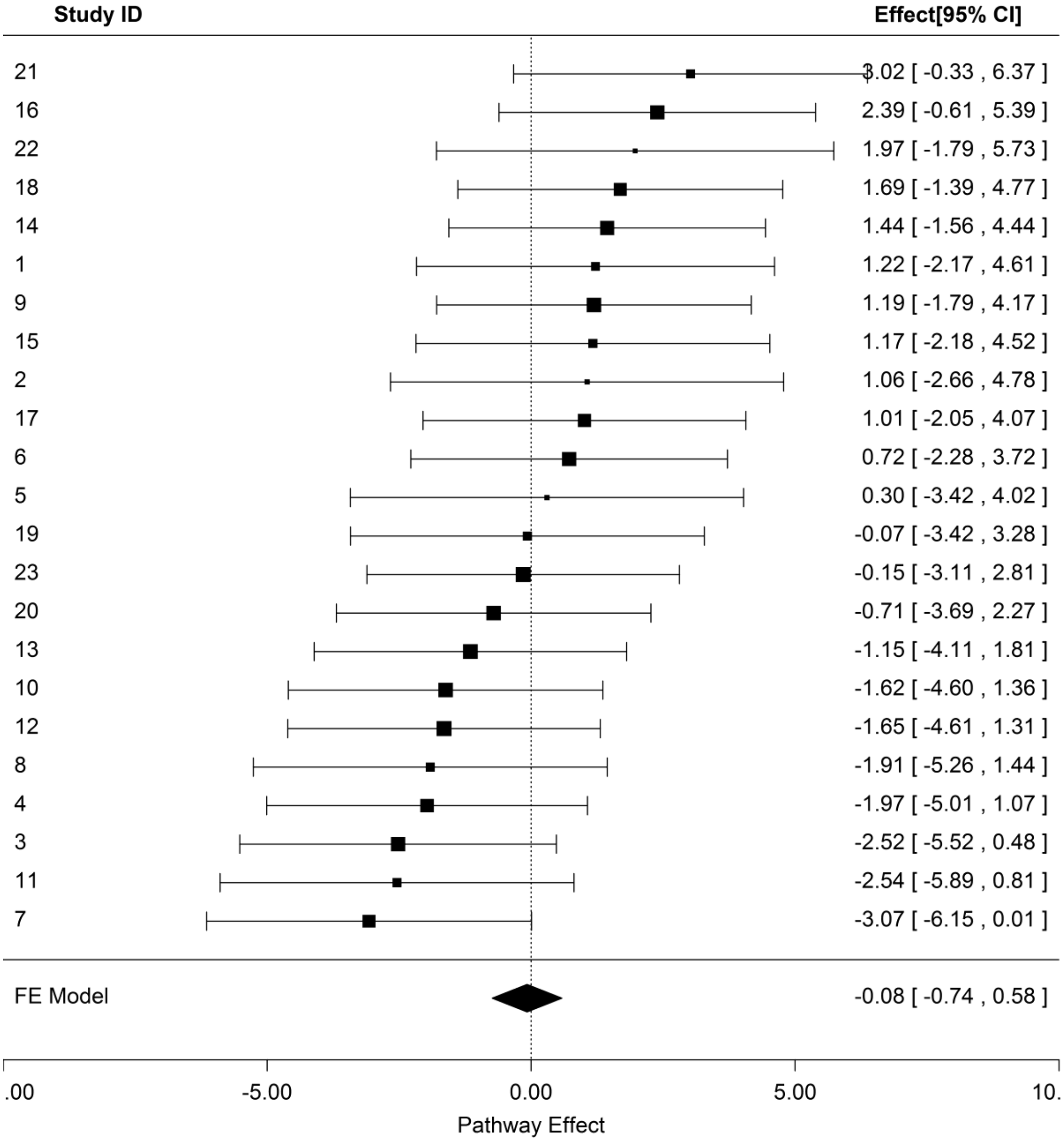

Supplementary Figure 4g. Meta-analysis of POU2F1-binding gene set (pid:1551)

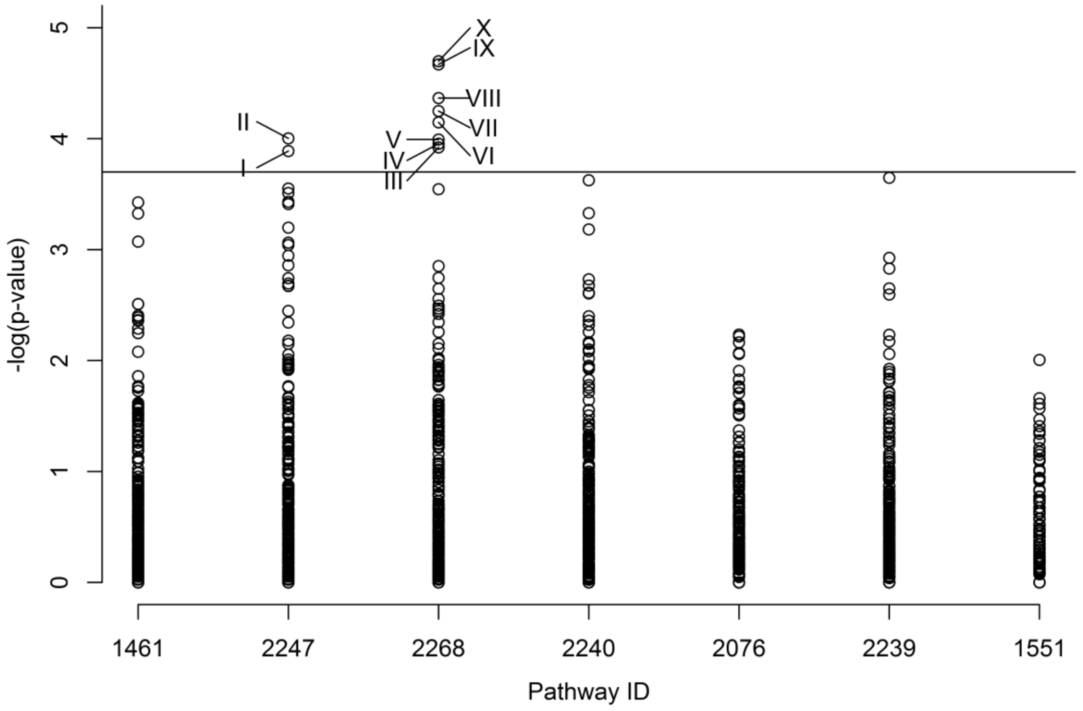

Supplementary Figure 5. Pairwise comparison of pathway expression patterns across studies. The X axis notes the pathway ID (PID) for the candidate gene sets, and the Y axis indicates the -$\log_{10}$ (*p-value*) for heterogeneity tests. The significant heterogeneity tests were (I): GDS Study 7 vs. 3; (II): GDS Study 7 vs. 23; (III): GDS Study 11 vs. 7; (IV): GDS Study 23 vs. 6; (V): GDS Study 23 vs. 17; (VI): GDS Study 11 vs. 6; (VII): GDS Study 11 vs. 21; (VIII): GDS Study 11 vs. 18; (IX): GDS Study 23 vs. 21; (X): GDS Study 11 vs. 17.
